# Draft Hybrid Genome Assembly of a Canadian Cyclospora cayetanensis Isolate

**DOI:** 10.1128/mra.01072-21

**Published:** 2022-02-03

**Authors:** Christine A. Yanta, Stephen M. J. Pollo, John R. Barta, Sarah J. Reiling, James D. Wasmuth, Brent R. Dixon, Rebecca A. Guy

**Affiliations:** a Division of Enteric Diseases, National Microbiology Laboratory at Guelph, Public Health Agency of Canada, Guelph, Ontario, Canada; b Department of Pathobiology, University of Guelph, Guelph, Ontario, Canada; c Department of Ecosystem and Public Health, Faculty of Veterinary Medicine, University of Calgary, Calgary, Alberta, Canada; d Bureau of Microbial Hazards, Food Directorate, Health Canada, Ottawa, Ontario, Canada; Universidad Nacional Autónoma de México

## Abstract

The apicomplexan parasite Cyclospora cayetanensis causes foodborne gastrointestinal disease in humans. Here, we report the first hybrid assembly for C. cayetanensis, which uses both Illumina MiSeq and Oxford Nanopore Technologies MinION platforms to generate genomic sequence data. The final genome assembly consists of 44,586,677 bases represented in 313 contigs.

## ANNOUNCEMENT

Cyclospora cayetanensis is an emerging human pathogen worldwide that causes the gastrointestinal disease cyclosporiasis. This coccidian parasite is becoming more prevalent in foodborne outbreaks across North America due to contaminated farm-produced crops imported from regions of endemicity (1). There are several inherent challenges in gathering genomic data for C. cayetanensis, including limited parasite material present in standard diagnostic samples and an inability to propagate the organism in a laboratory setting (2). The objective of this work was to generate the first whole-genome assembly from a C. cayetanensis specimen using both long- and short-read sequencing platforms. This hybrid approach will improve both the genome assembly available, similar to findings we described previously for Giardia intestinalis (3), and the identification of markers to refine current subtyping schemes to aid in outbreak investigations (4). We sequenced C. cayetanensis isolated from a positive human fecal specimen identified by the Newfoundland and Labrador Public Health Laboratory (NPHL) in 2020. Oocysts were purified from 5 g of stool as described previously (5) and incubated in 3% sodium hypochlorite for 10 min on ice prior to DNA extraction with the QIAamp Fast DNA Stool Mini Kit (Qiagen, Hilden, Germany). A bead beating step using lysing matrix Y (MP Biomedicals, OH, USA) at 6 m/s for 1 min on the Bead Ruptor 24 (Omni International, GA, USA) was included after the InhibitEx incubation to break the oocyst walls. The extracted DNA was purified using AMPure XP beads (Beckman Coulter, CA, USA). Two libraries were prepared with the Nextera DNA Flex kit and rapid PCR barcoding kit (SQK-RPB004) using 1 ng of purified DNA as the starting material. The resulting libraries were sequenced on the MiSeq platform (Illumina, Inc., CA, USA) using the v3 reagent kit (600 cycles) with a 2 × 300-bp paired-end protocol and on the MinION platform (Oxford Nanopore Technologies, Oxford, UK) using the flow cell type R9.4.1 (FLO-MIN106D). Sequencing on the Illumina MiSeq system yielded 11 million paired-end reads (5.2 Gbp), and sequencing on the Oxford Nanopore Technologies MinION system generated 1.0 million reads (2.9 Gbp), with an average fragment length of 3.3 kbp. The quality of the raw short reads was examined using FastQC (6), and the reads were filtered and trimmed using BBDuk v37.62 from the BBTools suite (7). Long reads were base called using Oxford Nanopore Technologies Guppy v4.0.11, and reads with average quality scores of <7 were removed. The resulting MiSeq short reads and MinION long reads were *de novo* assembled using hybridSPAdes v3.11.1 (8, 9) using default parameters. A similarity search using BLAST v2.9.0+ (10) was performed against the RefSeq genome database for coccidian parasites with the initial assembly, and all contaminating contigs were removed. Polishing of the assembly was performed by mapping the short and long reads back to the assembly with Minimap2 v2.17-r941 (11) and correcting them with Pilon v1.23 (12). The final assembly is 313 contigs, with a total genome size of 44,586,677 bases (the largest contig is 1,973,156 bases), a G+C content of 51.9%, an *N*_50_ value of 526,712 bases, and an *L*_50_ value of 24 contigs. Based on these statistics, this is the most continuous genome assembly described to date of the 37 draft genomes available for C. cayetanensis.

### Data availability.

This whole-genome shotgun project has been deposited in DDBJ/EMBL/GenBank under the accession no. JAJEWR000000000. The version described in this paper is the first version, JAJEWR010000000. Raw sequence reads are available under the BioProject no. PRJNA772675.
